# Supplementary material for: Cryo-EM reveals the architecture of the PELP1-WDR18 molecular scaffold
Source: Nat Commun. 2022 Nov 9;13:6783. doi: 10.1038/s41467-022-34610-0 (PMC9646879; doi:10.1038/s41467-022-34610-0)
Supplement: Supplementary file 1 — Supplementary Information [file 41467_2022_34610_MOESM1_ESM.pdf]

Supplementary Information

Cryo-EM Reveals the Architecture of the PELP1-WDR18 Molecular Scaffold

Jacob Gordon[1,2,3,4], Fleur L. Chapus[5], Elizabeth G. Viverette[6], Jason G. Williams[5], Leesa J. Deterding[5], Juno M. Krahn[6], Mario J. Borgnia[6], Joseph Rodriguez[5], Alan J. Warren[2,3,4], and Robin E. Stanley[1*]

[1]Signal Transduction Laboratory, National Institute of Environmental Health Sciences, National Institutes of Health, Department of Health and Human Services, 111 T. W. Alexander Drive, Research Triangle Park, NC 27709, USA.

[2]Cambridge Institute for Medical Research, Cambridge Biomedical Campus, Keith Peters Building, Hills Rd, Cambridge, CB2 0XY, UK.

[3]Wellcome Trust-Medical Research Council Stem Cell Institute, Jeffrey Cheah Biomedical Centre, Puddicombe Way, Cambridge Biomedical Campus, Cambridge, CB2 0AW, UK.

[4]Department of Haematology, University of Cambridge School of Clinical Medicine, Jeffrey Cheah Biomedical Centre, Puddicombe Way, Cambridge Biomedical Campus, Cambridge, CB2 0AW, UK.

[5]Epigenetics and Stem Cell Biology Laboratory, National Institute of Environmental Health Sciences, National Institutes of Health, Department of Health and Human Services, 111 T. W. Alexander Drive, Research Triangle Park, NC 27709, USA.

[6]Genome Integrity and Structural Biology Laboratory, National Institute of Environmental Health Sciences, National Institutes of Health, Department of Health and Human Services, 111 T. W. Alexander Drive, Research Triangle Park, NC 27709, USA.

*Correspondence and requests for materials should be addressed to (email: robin.stanley@nih.gov)

This PDF file includes:

Supplementary Figures 1 to 11
Supplementary Notes 1 to 2
Supplementary Table 1

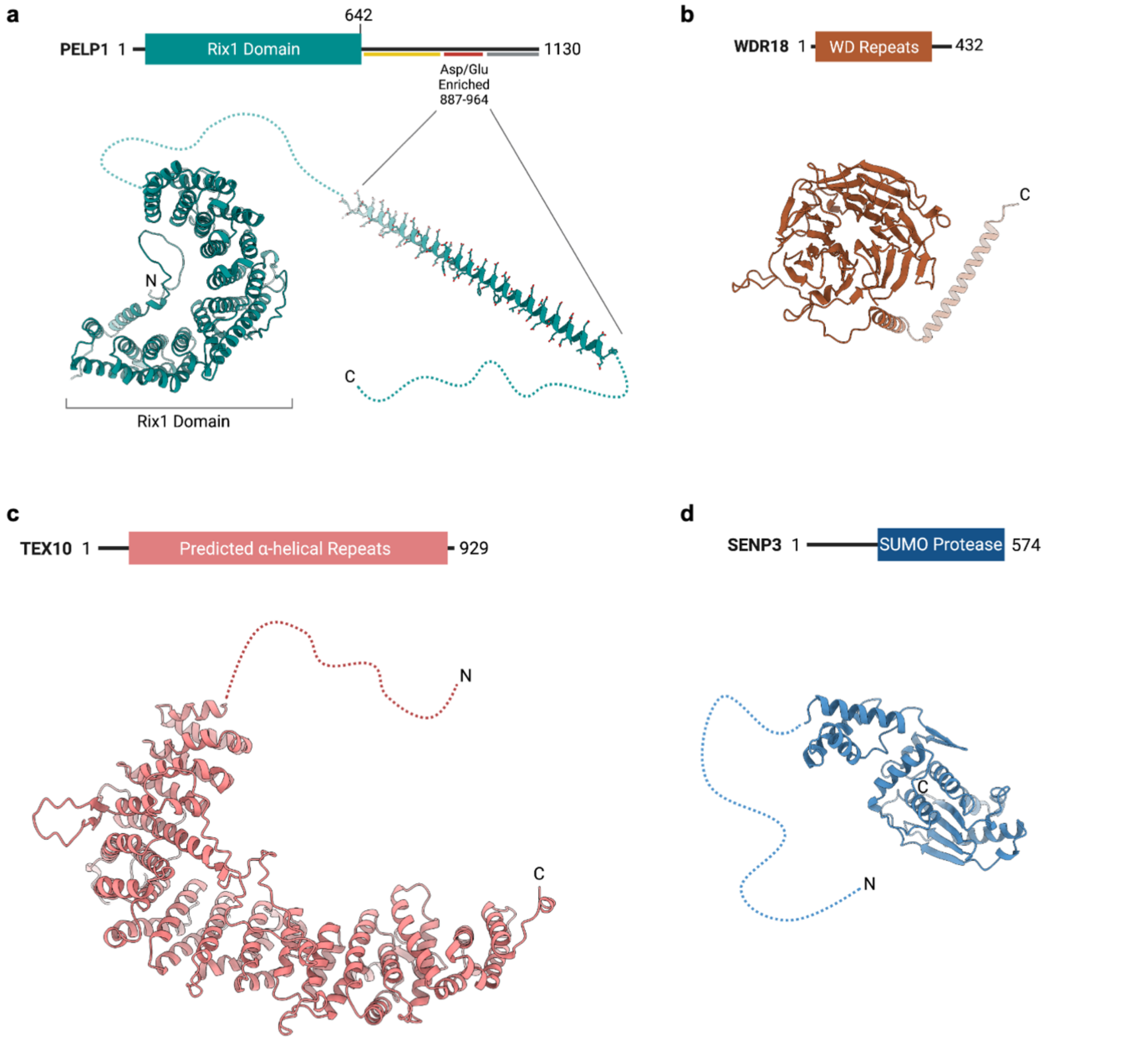

Supplementary Fig. 1: **Alphafold structural predictions of individual human Rix1 complex members[1,2]. a** UniProt Q8IZL8 corresponding to human PELP1. The N-terminal Rix1 domain of this predicted model was used as a modeling starting point for PELP1 Rix1 domain experimental cryo-EM data. Discorded C-terminal region is mostly omitted for clarity and depicted by dashed lines. The Glu enriched region of PELP1 is predicted to form a large helix secondary structure. **b** UniProt Q9BV38 corresponding to human WDR18. This predicted model was used as a modeling starting point for WDR18 experimental cryo-EM data. **c** UniProt Q9NXF1 corresponding to human TEX10. Most of this protein is predicted to be a helical-repeat scaffold. **d** UniProt Q9H4L4 corresponding to human SENP3. N-terminal disordered region is omitted for clarity and depicted by dashed line.

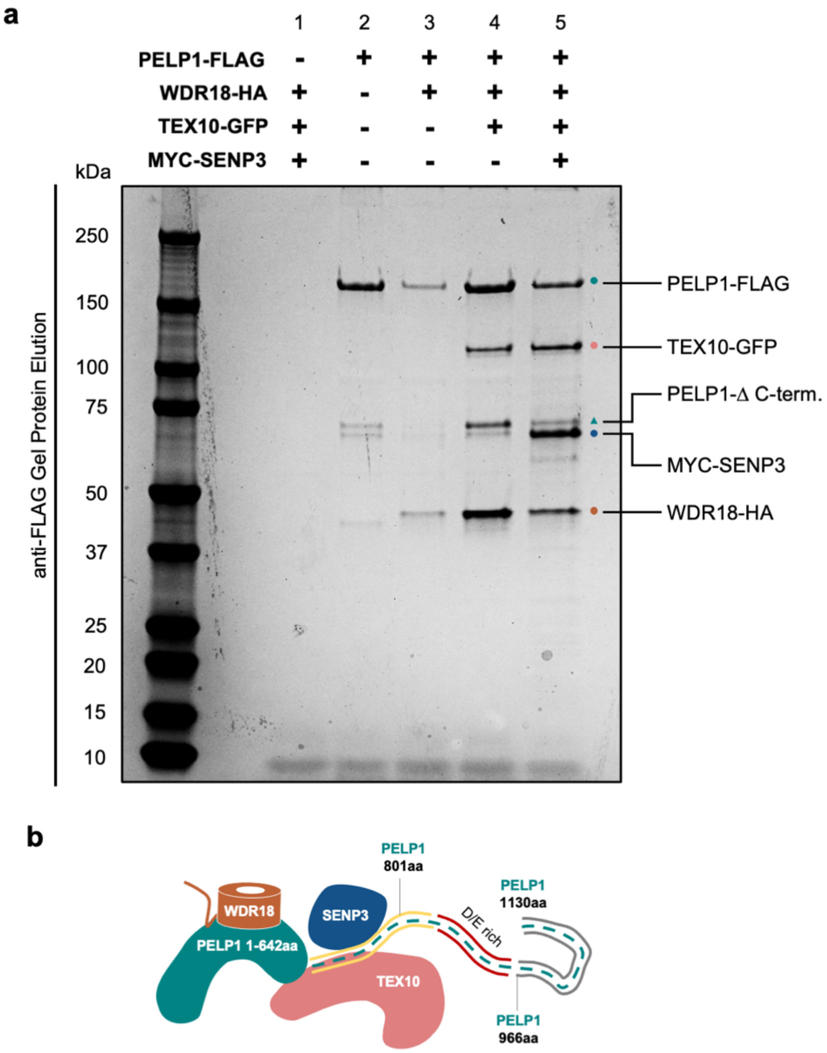

Supplementary Fig. 2: C-terminal FLAG tagged PELP1 IP of sequentially built Rix1 complexes. a The identical experimental designed used in Fig. 1b was repeated with C-terminal FLAG tagged PELP1 as bait to enrich for full-length PELP1 (1-1130aa). Eluted complexes were subjected to SDS-PAGE and total protein staining. Better enrichment of full-length PELP1 was observed along with continued presence of C-terminal truncated PELP1 (band indicated by teal triangle) indicating a likely higher-order stoichiometry. Association of TEX10 and SENP3 appeared to stabilize the C-terminal region of PELP1 and facilitate stoichiometric assembly of the complex (lanes 4 and 5). Co-IP experiment in (**a**) has been performed at least three times with reproducible results. **b** Cartoon representation of human Rix1 complex members interacting with proteins/regions determined in (**a**) and main Fig. 1b.

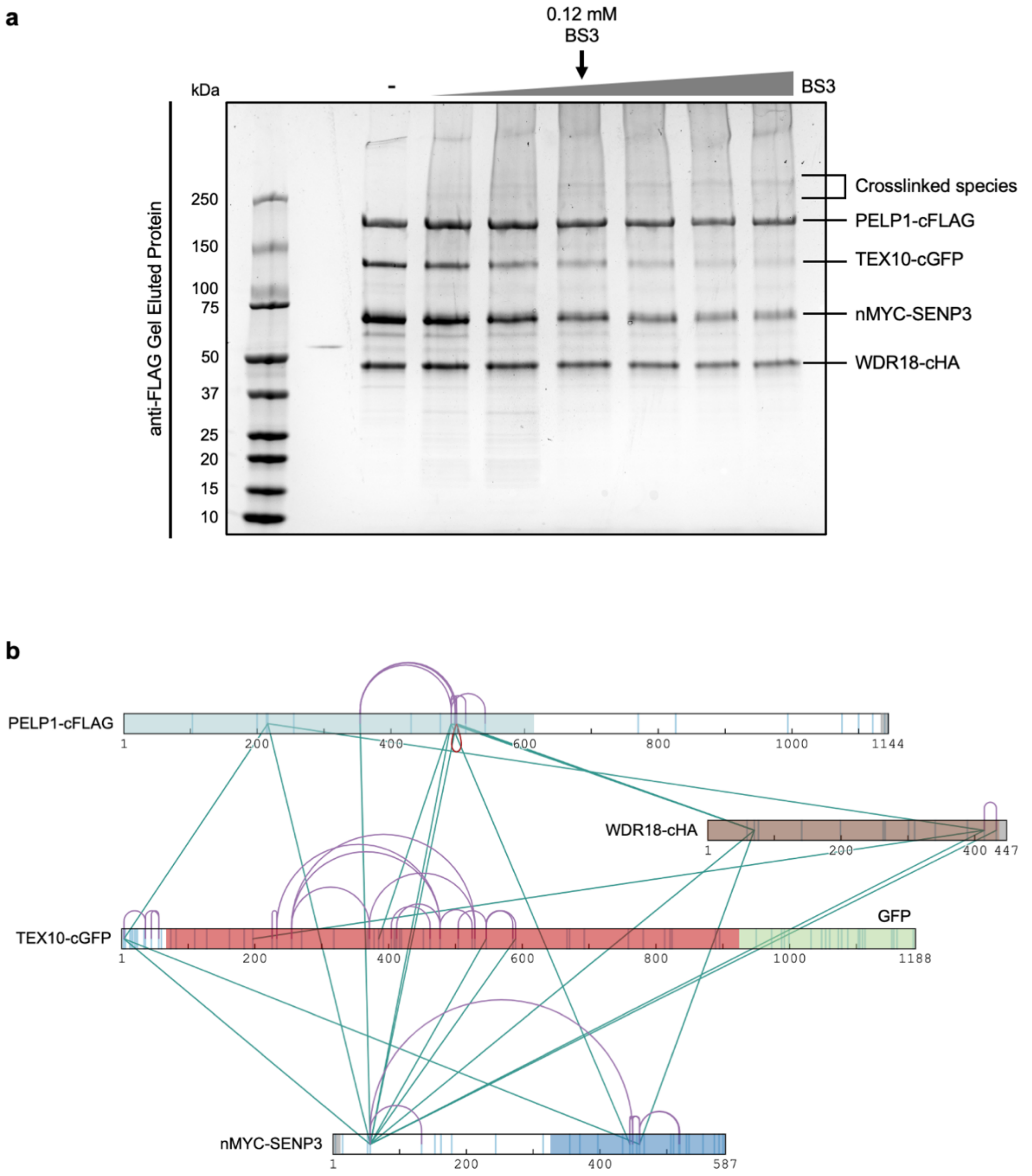

Supplementary Fig. 3: BS3 crosslinking mass spectrometry of the human Rix1 complex. a The human Rix1 complex was isolated using C-terminally FLAG tagged PELP1 as bait, concentrated, and treated with increasing amounts of BS3. These samples were subjected to SDS-PAGE and total protein staining to assess for protein complexes covalently crosslinked. Arrow indicates the concentration of BS3 used for further crosslinking experiments and mass spectrometry analysis. Co-IP experiment in (**a**) has been performed at least three times with reproducible results. BS3 X-linking optimization was performed twice before mass spectrometry analysis. **b** Depiction of experimentally determined crosslinks between the Rix1 complex members. Predicted ordered regions of the proteins are color shaded. Amino acid boundaries are labeled below each protein. Green lines between proteins illustrate inter-molecular crosslinks in the Rix1 complex. Purple arches within individual proteins illustrate intra-molecular protein crosslinks. Red arches indicate inter-molecular crosslinks between PELP1-PELP1 protomers likely in separate complex assemblies (based on lysine position in PELP1-WDR18 structure). Crosslinks detected on epitope tags were omitted for clarity. Crosslink illustration was done with xiNET[3].

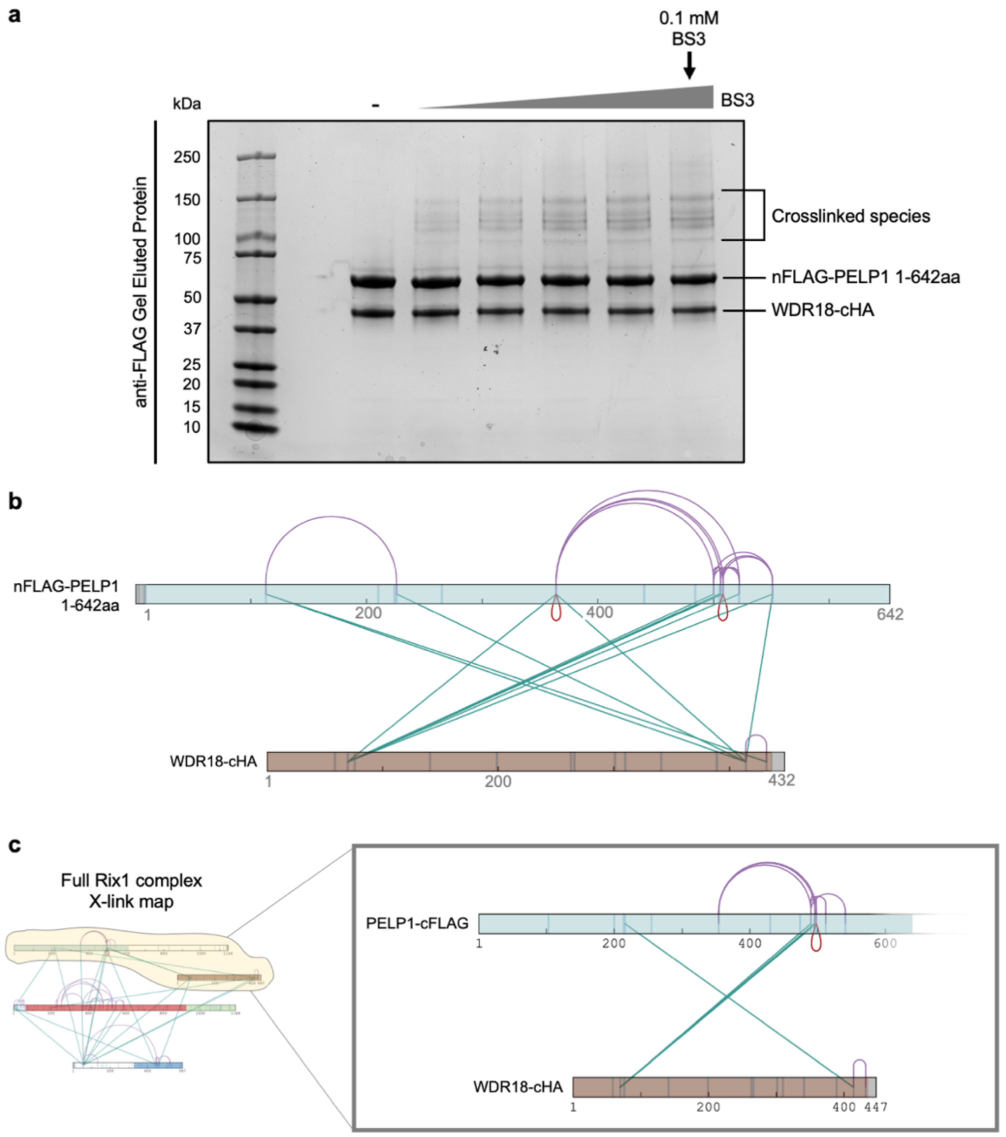

Supplementary Fig. 4: BS3 crosslinking mass spectrometry of the human PELP1 Rix1 domain - WDR18 sub-complex. a The PELP1 Rix1 domain bound to WDR18 was isolated using N-terminally FLAG tagged PELP1 Rix1 domain as bait, concentrated, and treated with increasing amounts of BS3. These samples were subjected to SDS-PAGE and total protein staining to assess for protein complexes covalently crosslinked. Arrow indicates the concentration of BS3 used for further crosslinking experiments and mass spectrometry analysis. Co-IP experiment in (**a**) has been performed at least three times with reproducible results. BS3 X-linking optimization was performed twice before mass spectrometry analysis. **b** Depiction of experimentally determined crosslinks between PELP1 Rix1 domain and WDR18. Predicted ordered regions of the proteins are color shaded. Amino acid boundaries are labeled below each protein. Predicted ordered regions of the proteins are color shaded. Green lines illustrate inter-molecular crosslinks between PELP1 Rix1 domain and WDR18. Purple arches within individual proteins illustrate intra- and inter-molecular PELP1-PELP1 and WDR18-WDR18 protein crosslinks. Red arches indicate inter-molecular crosslinks between PELP1-PELP1 protomers likely in separate sub-complex assemblies (based on lysine position in PELP1-WDR18 structure). Crosslinks detected on PELP1's N-terminal FLAG tag were omitted for clarity. Crosslink illustration was done with xiNET[3]. **c** Reference of full Rix1 complex crosslinking data with zoom-in of crosslinks observed between PELP1 Rix1 domain and WDR18. Identical crosslinks in these protein regions are observed between the two crosslinking datasets.

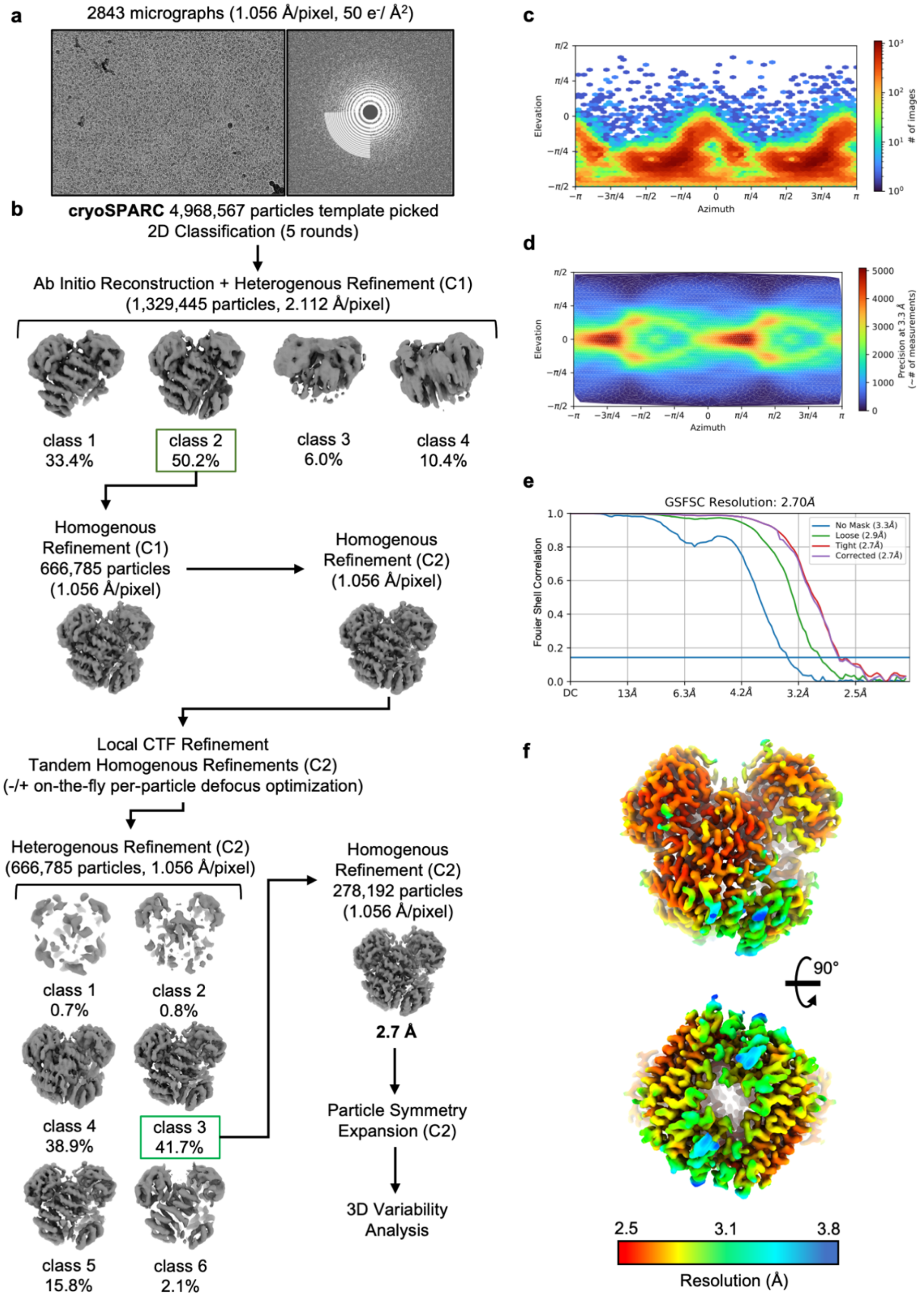

Supplementary Fig. 5: Overview of cryo-EM processing scheme for human PELP1-WDR18 sub-complex. a A representative micrograph of PELP1-WDR18 in vitreous ice and the corresponding power spectrum collected on a Titan Krios. **b** Cryo-EM image processing workflow**.** Particle images were picked from 2843 collected micrographs from a gold coated holey carbon grid. Image processing and 3D refinements resulted in a 2.7 Å consensus map used for molecular modeling and further 3D variability analysis[4]. **c** Angular distribution of PELP1-WDR18 particles. **d** Posterior precision directional distribution of PELP1-WDR18 particles. **e** Fourier shell correlation (FSC) curve for PELP1-WDR18 reconstruction. The overall resolution is 2.63 according to the FSC 0.143 criteria[5,6]. **f** Cryo-EM reconstruction of PELP1-WDR18 colored based on local resolution calculated using cryoSparc v2[7].

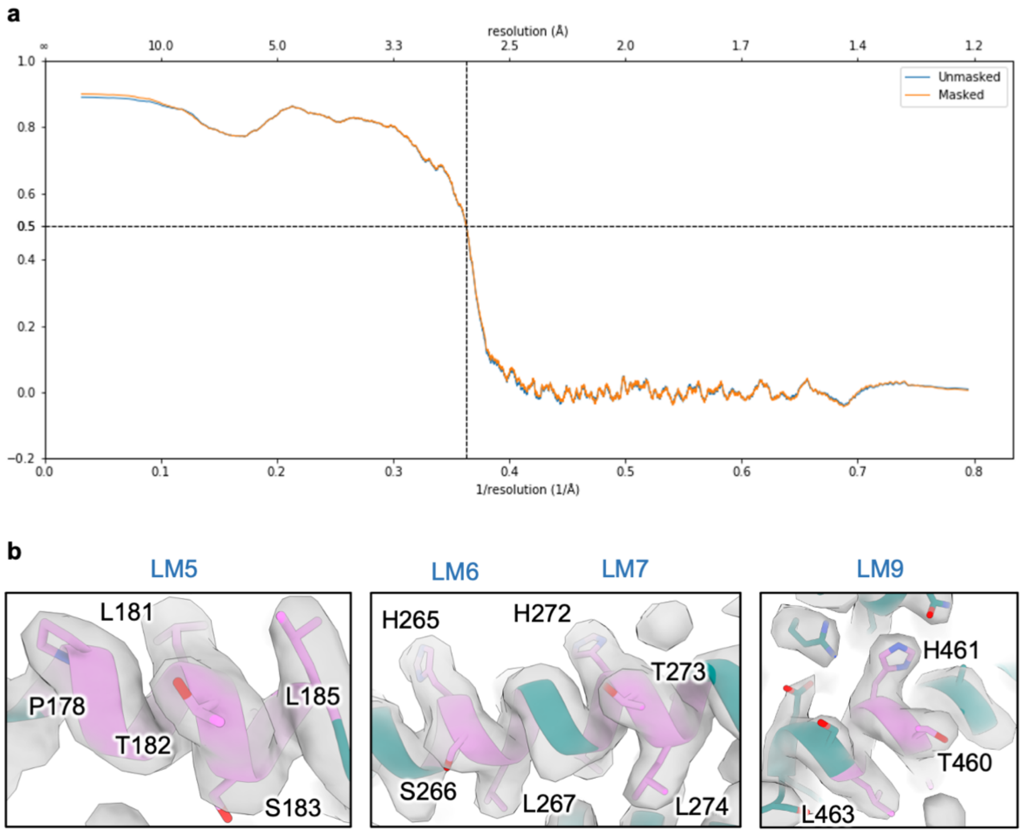

Supplementary Fig. 6: FSC map vs. model summary plot and residue density fits. a FSC map vs. model plot generated in PHENIX during model validation and Molprobity statistics[8,9]. **b** Representative map densities showing modeled residue fits. The four solvent exposed LxxLL motifs in PELP1's Rix1 domain (LM5, LM6, LM7, LM9) were chosen as examples.

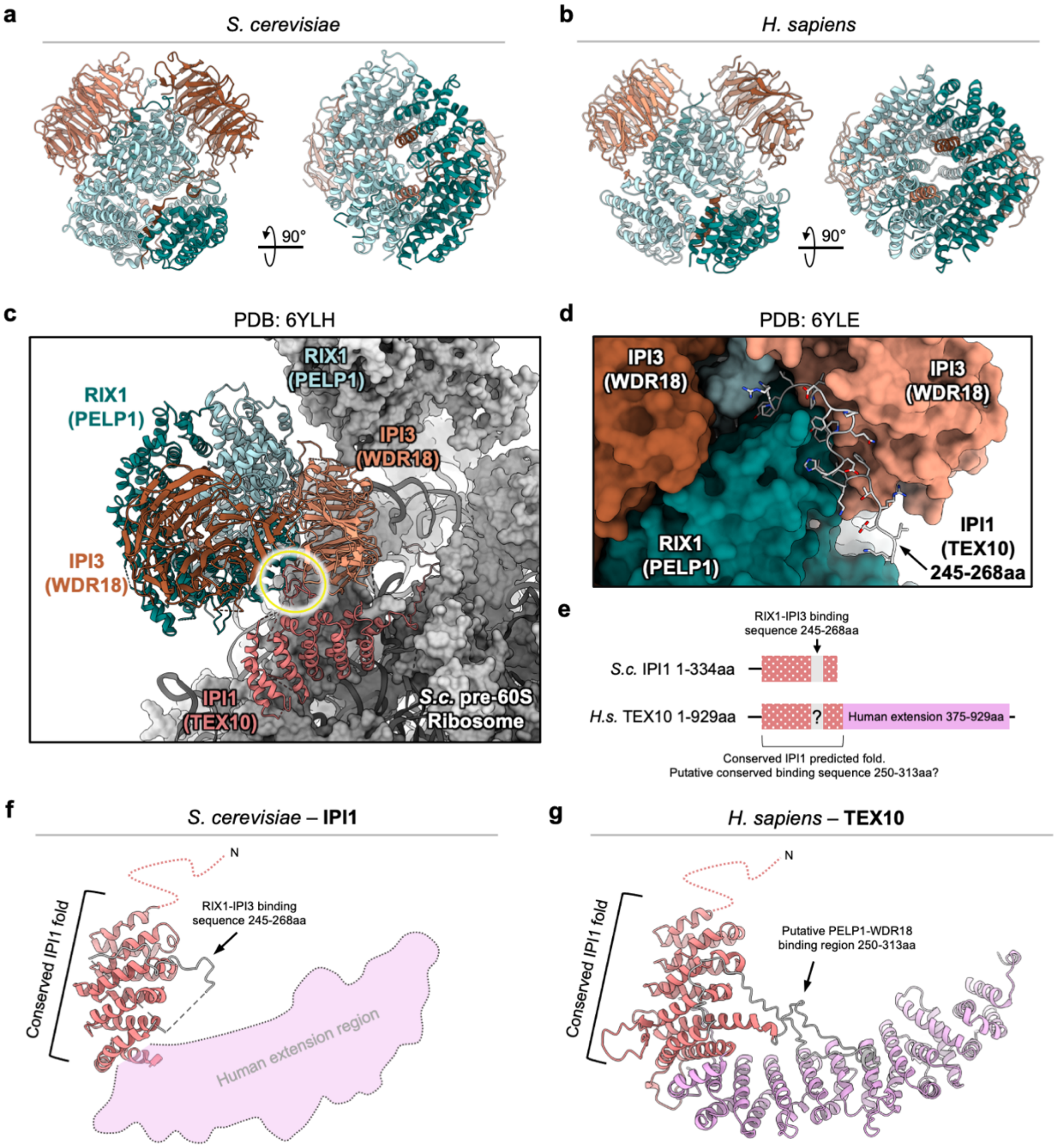

Supplementary Fig. 7: The conserved architecture of yeast and human PELP1-WDR18 sub-complexes is biologically relevant for pre-60S ribosome and IPI1/TEX10 association.

a Model of ribosome bound yeast Rix1 sub-complex assembly RIX1-IPI3 (PDB: 6YLE)[10]. **b** Model of human PELP1-WDR18 assembly from this study. Homologous protomers match same color scheme as in yeast assembly (a). **c** Experimental model of the yeast Rix1 complex RIX1-IPI3-IPI1 bound to the pre-60S ribosome in *S. cerevisiae* (PDB: 6YLH)[10]. This structural data supports the biological relevance and conservation of the PELP1-WDR18 (RIX1-IPI3) heterotetramer. IPI1 (TEX10) interacts directly with the pre-60S ribosome and acts as a platform for RIX1-IPI3 to bind through a small interface circled in yellow. **d** Zoom-in of the binding interface between IPI1 (TEX10) and RIX1-IPI3 (PELP1-WDR18). **e** Cartoon comparisons of the IPI1/TEX10 homologous proteins exhibiting a conserved N-terminal fold corresponding to the yeast IPI1 structure. Human TEX10 contains a large C-terminal extension region (pink color) tripling the size of yeast IPI1. **f** Structure of individual yeast IPI1 protomer with its conserved fold and the TEX10 homolog extension region depicted. **g** AlphaFold predicted structure of the human TEX10 protomer (UniProt Q9NXF1) showing the conserved IPI1 fold in the N-terminus and a unique extension region (pink) that is predicted to be structured with α-helices. A putative PELP1-WDR18 binding region in a disordered loop is predicted to be conserved in TEX10 when structurally aligned with yeast IPI1.

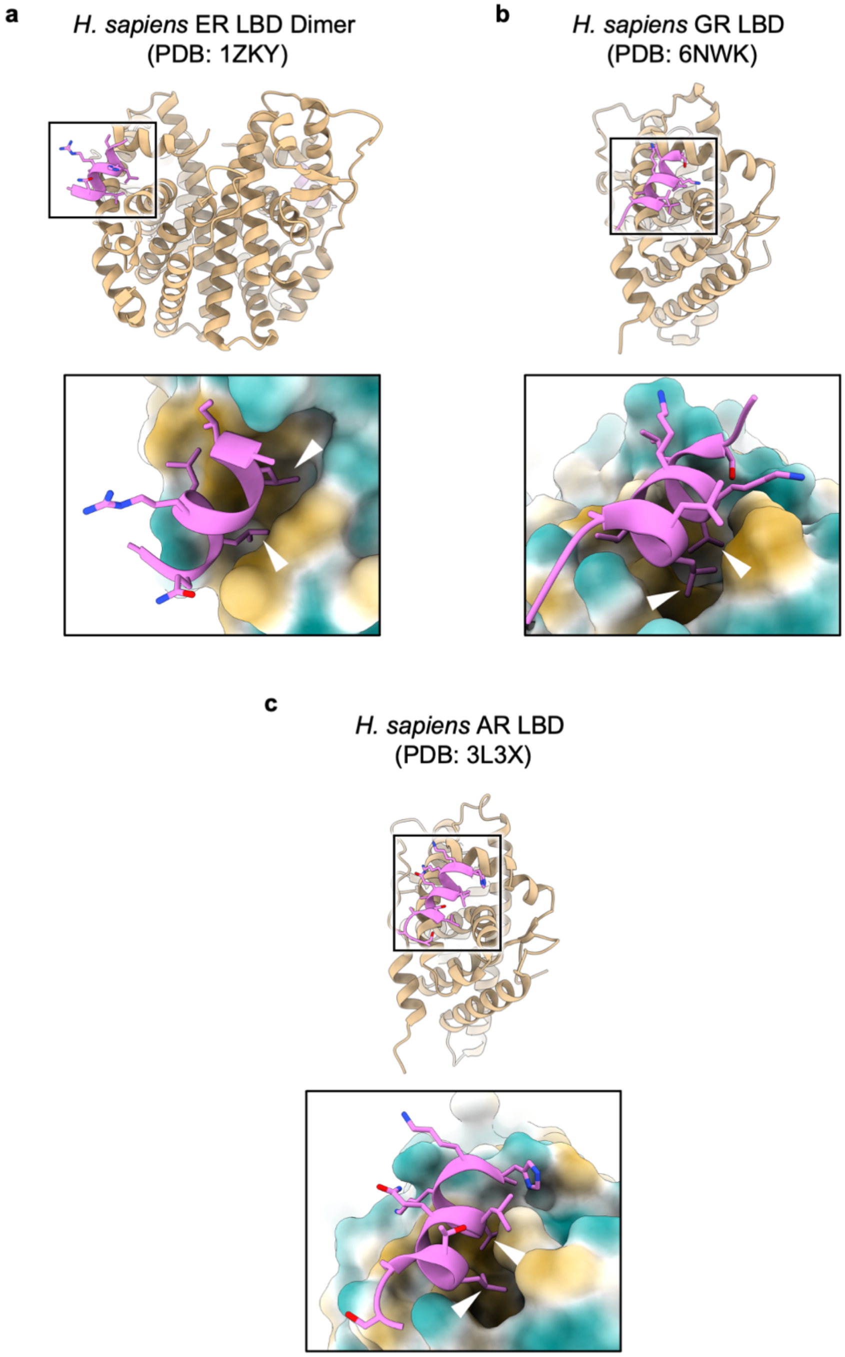

Supplementary Fig. 8: Experimental structures of LxxLL co-activator motifs bound to the AF-2 domain of various human steroid receptors. a Structure of human estrogen receptor (ER) ligand binding domain (LBD) dimerized and bound to LxxLL motif (magenta)[11]. Zoom of LxxLL motif bound to AF-2 hydrophobic pocket is below structure. LBD is visualized with a surface representation depicting hydrophobicity (light brown = non-polar, hydrophobic surface) calculated in ChimeraX[12]. White arrows denote invariant leucine residues that bury into the hydrophobic AF-2 co-activator binding pocket and are critical for the binding mechanism. **b** Structure of human glucocorticoid receptor (GR) LBD bound to LxxLL motif[13]. Illustration matches that of ER in (**a**). **c** Structure of human androgen receptor (AR) LBD bound to LxxLL motif[14]. Illustration matches that of ER and GR in (**a**,**b**).

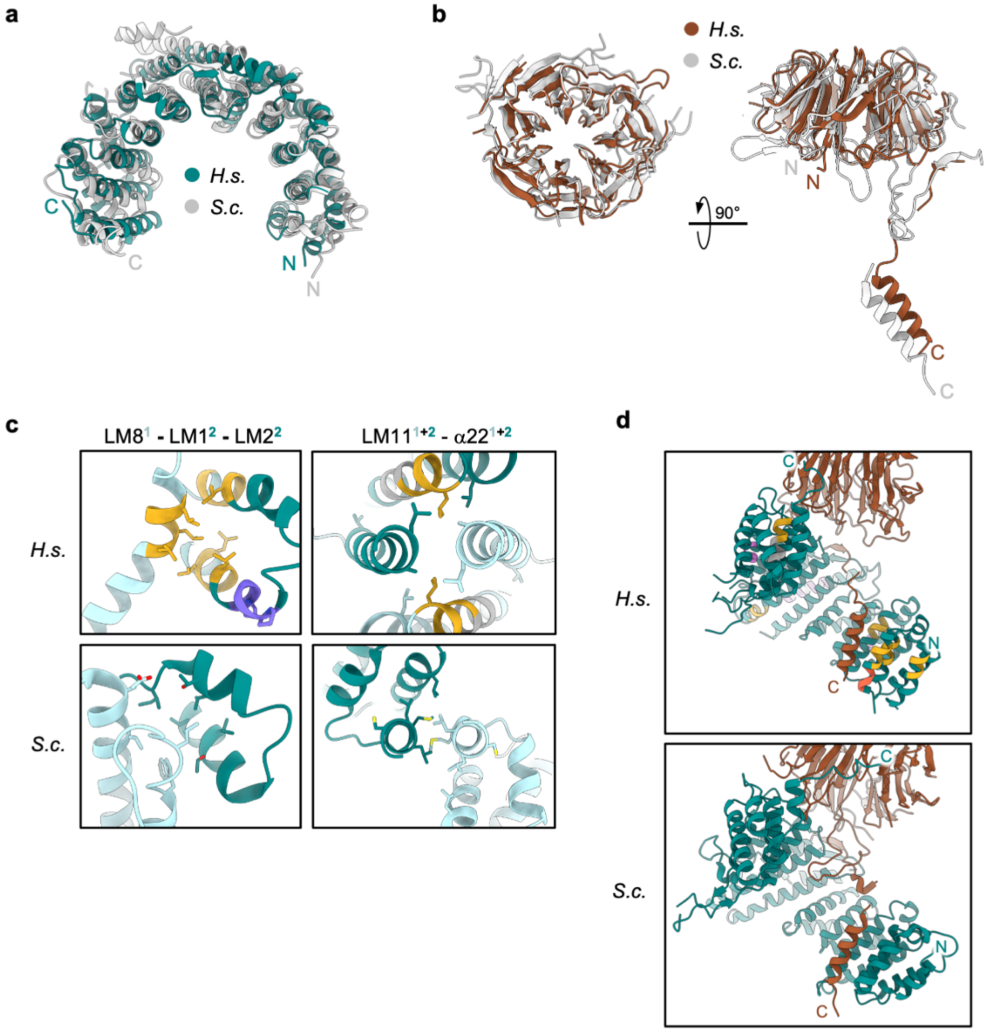

Supplementary Fig. 9: Structural comparisons of human and yeast PELP1/WDR18 protomers and sub-complex interaction interfaces. a PELP1 Rix1 domain protomer overlay with yeast RIX1 (PDB: 6YLE) showing similar α-helical solenoid structure that is conserved. **b** WDR18 protomer overlay with yeast IPI3 (PDB: 6YLE) showing conserved β-propeller domain and C-terminal tail regions. **c** Comparison of the conserved PELP1 Rix1 domain dimerization interfaces in humans and yeast. LxxLL and PxxP motifs are colored accordingly in human interfaces. Analogous interfaces from yeast are shown below human. **d** Comparison of the conserved PELP1 Rix1 domain interaction with WDR18 facilitated through the WDR18 C-terminal tail and capping by the β-propeller domain. Binding motifs are colored accordingly in human PELP1. Abbreviations are as follows: *Homo sapiens* (H.s.), *Saccharomyces cerevisiae* (S.c.).

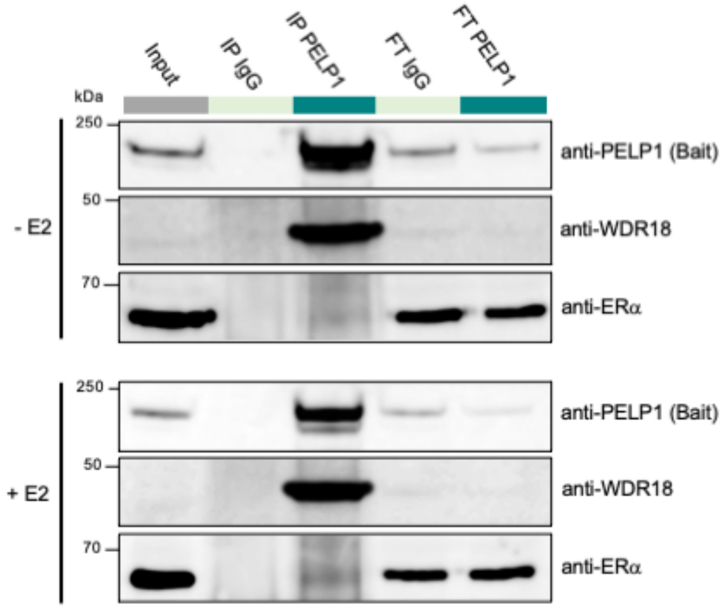

Supplementary Fig. 10: Co-IP of endogenous PELP1 from MCF7 cells. MCF7 cells treated with either 10 nM E2 or ethanol for 24 hours were lysed and endogenous PELP1 co-IP'd with anti-PELP1 antibody. Control IgG and IP samples were subject to SDS-PAGE and Western blot to detect endogenous proteins associated with PELP1. PELP1 was enriched in the IP lane along with WDR18. We could not detect robust amounts of ERα in the endogenous PELP1 IP lane for both +/- E2 treatment. co-IP and Western blot experiment in (**a**) was performed three times with reproducible results. Uncropped gel images available in the Source Data file.

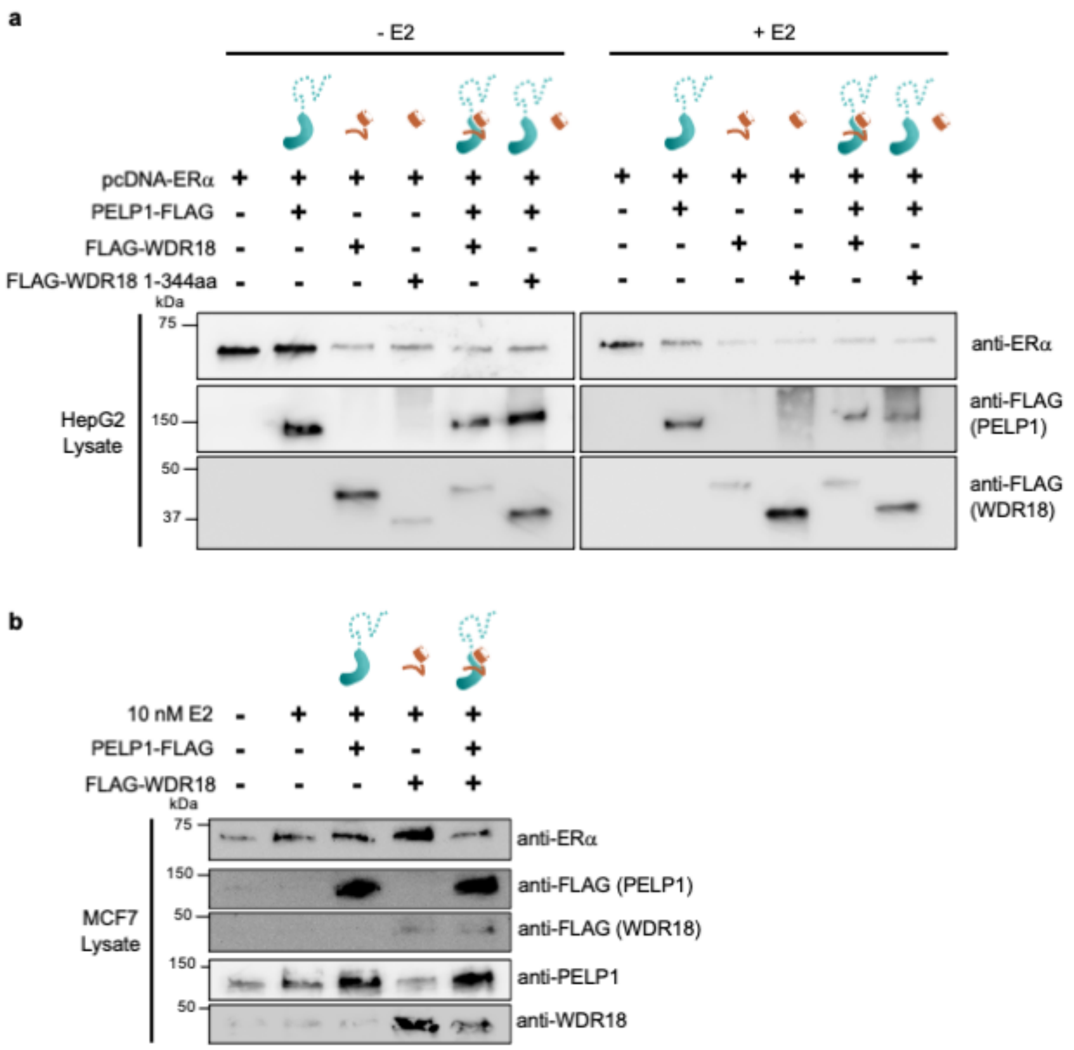

Supplementary Fig. 11: Western blots of exogenous and endogenous proteins of interest corresponding to HepG2 and MCF7 3X ERE luciferase assays. **a** Western blot of exogenously expressed proteins in the HepG2 3X ERE reporter assay in main figure 6b. Untagged ERα and FLAG tagged PELP1 and WDR18 constructs' expression was detected in HepG2 cell lysate from the luciferase reporter assay experiment. **b** Western blot of exogenously and endogenously expressed proteins in the MCF7 3X ERE reporter assay in main figure 6c. FLAG tagged PELP1 and WDR18 constructs' expression was detected in MCF7 cell lysate from the luciferase reporter assay experiment. PELP1 and WDR18 antibodies detecting endogenous proteins were also used to detect exogenous protein expression over the endogenous levels. Western blots in (**a**,**b**) from luciferase assays were performed on two of the experimental replicates from Fig. 6b,c. Representative blots are shown. Uncropped gel images available in the Source Data file.

a

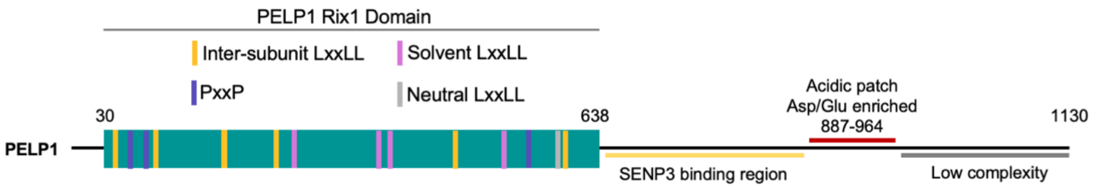
PELP1 Rix1 Domain
Inter-subunit LxxLL
Solvent LxxLL
PxxP
Neutral LxxLL
30
638
Acidic patch
Asp/Glu enriched
887-964
1130
PELP1
SENP3 binding region
Low complexity

b

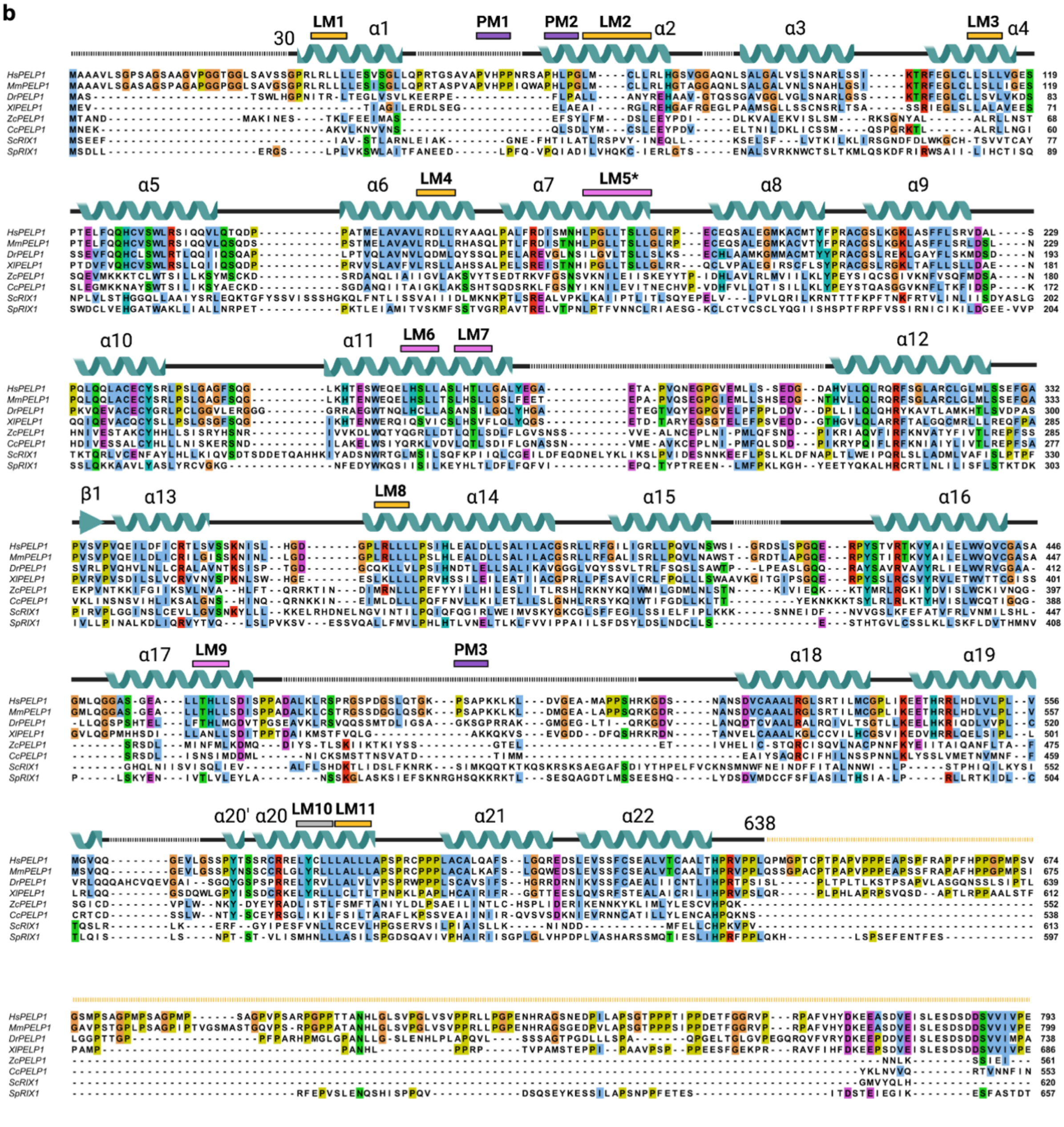
30
LM1
α1
PM1
PM2
LM2
α2
α3
LM3
α4
α5
α6
LM4
α7
LM5*
α8
α9
α10
α11
LM6
LM7
α12
β1
α13
LM8
α14
α15
α16
α17
LM9
PM3
α18
α19
α20'
α20
LM10
LM11
α21
α22
638
HsPELP1
MmPELP1
DrPELP1
XlPELP1
ZcPELP1
CcPELP1
ScRIX1
SpRIX1

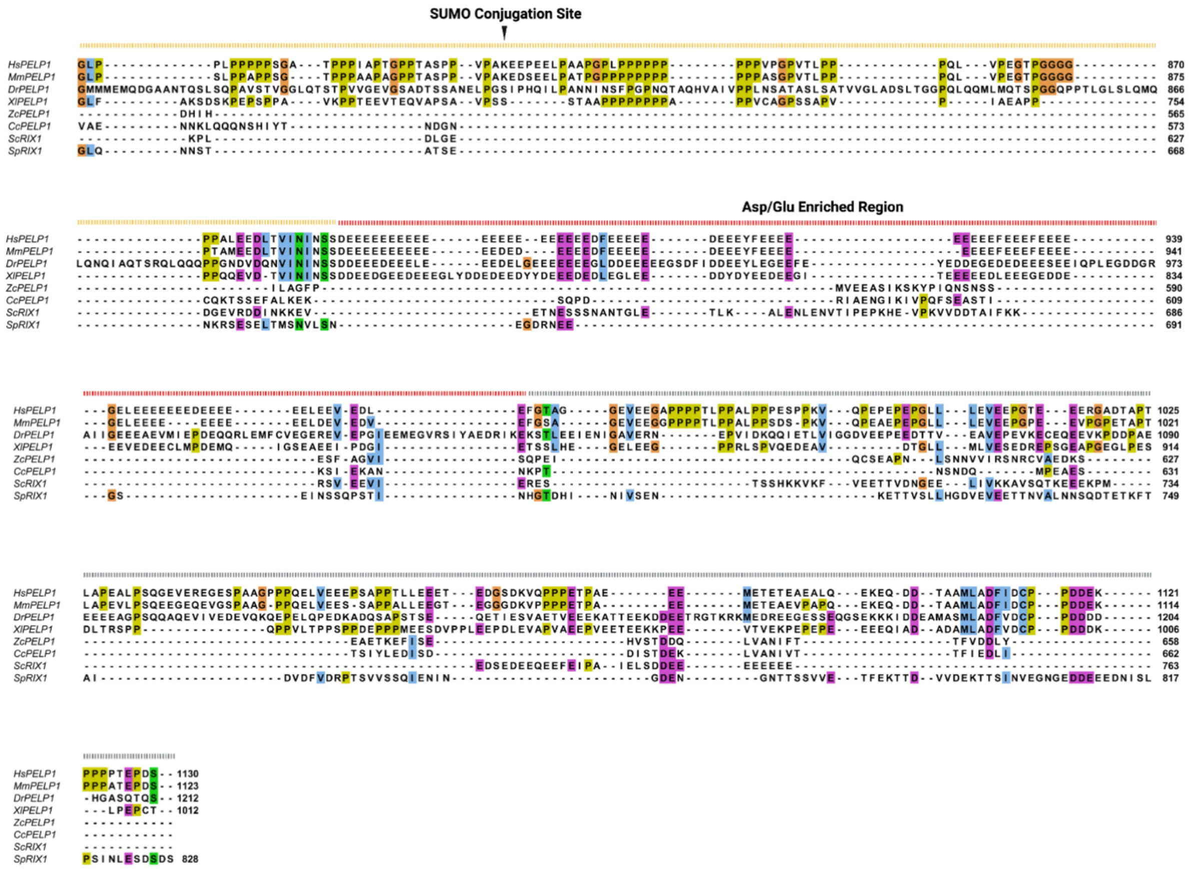

Supplementary Note 1: PELP1 multiple amino acid sequence alignment. a Schematic of PELP1 domain boundaries and labeled features. **b** Sequence alignment for PELP1. Alignment was performed with PROMALS3D[15] and illustrated in JalView[16]. Secondary structure determined experimentally in this study and select chemical features/motifs are included above the sequence. Asterisk on LM5 denotes an overlapping LxxLL sequence register. Abbreviations are as follows: *Homo sapiens* (Hs), *Mus musculus* (Mm), *Danio rerio* (Dr), *Xenopus laevis* (Xl), *Zeugodacus cucurbitae* (Zc), *Ceratitis capitata* (Cc), *Saccharomyces cerevisiae* (Sc), *Schizosaccharomyces pombe* (Sp).

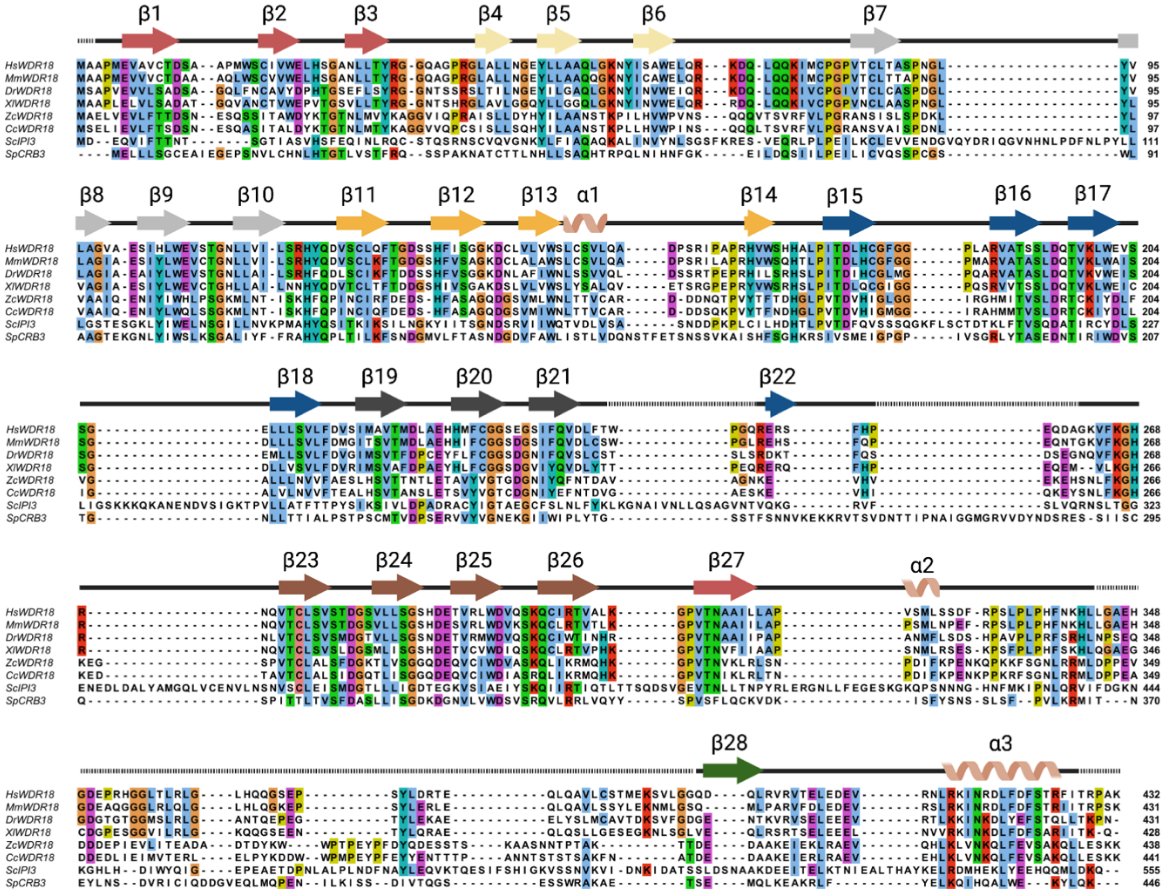

Supplementary Note 2: WDR18 multiple amino acid sequence alignment. Sequence alignment for WDR18. Alignment was performed with PROMALS3D[15] and illustrated in JalView[16]. Secondary structure determined experimentally in this study are included above the sequence. Like-colored arrows correspond to β-strands within the same β-sheet of WDR18's β-propeller domain. Abbreviations are as follows: *Homo sapiens* (Hs), *Mus musculus* (Mm), *Danio rerio* (Dr), *Xenopus laevis* (Xl), *Zeugodacus cucurbitae* (Zc), *Ceratitis capitata* (Cc), *Saccharomyces cerevisiae* (Sc), *Schizosaccharomyces pombe* (Sp).

Expression Vector	Gene	Source
pcDNA3.1-nDYK	Pelp1 WT (1-1130aa)	GenScript
pcDNA3.1-cDYK	Pelp1 WT (1-1130aa)	GenScript
**pcDNA3.1-nDYK**	**Pelp1 1-642aa**	GenScript
**pcDNA3.1-cHA**	**Wdr18 WT (1-432aa)**	GenScript
pcDNA3.1-cDYK	PELP1 (1-966aa)	This work
pcDNA3.1-cDYK	PELP1 (1-801aa)	This work
pcDNA3.1-cDYK	PELP1 (1-642aa)	This work
pcDNA3.1-nDYK	PELP1 (643-1130aa)	This work
pcDNA3.1-nDYK	Wdr18 WT (1-432aa)	This work
pcDNA3.1-nDYK	Wdr18 1-344aa	This work
pLexM-cGFP	Tex10 WT (1-929aa)	This work
pcDNA3.1-nMYC	Senp3 WT (1-574aa)	This work
pLexM-nGFP	Senp3 WT (1-574aa)	This work
pcDNA3.1-DEST	Empty	Li, Y. et al.
pcDNA3.1-DEST	ER alpha WT (1-595aa)	Li, Y. et al.
pGL3	TATA - 3xERE – Firefly Luc.	Li, Y. et al.
pRL-TK	Ranilla Luc.	Li, Y. et al.

Supplementary Table 1: Mammalian protein expression and dual luciferase assay plasmids used in this study. Plasmids in bold denote their use for PELP1-WDR18 expression and sample preparation for cryo-EM analysis. Luciferase reporter assay reagents from work cited[17].

Supplementary References

1 Jumper, J. *et al.* Highly accurate protein structure prediction with AlphaFold. *Nature* **596**, 583-589, doi:10.1038/s41586-021-03819-2 (2021).

2 Varadi, M. *et al.* AlphaFold Protein Structure Database: massively expanding the structural coverage of protein-sequence space with high-accuracy models. *Nucleic Acids Res* **50**, D439-D444, doi:10.1093/nar/gkab1061 (2022).

3 Combe, C. W., Fischer, L. & Rappsilber, J. xiNET: cross-link network maps with residue resolution. *Mol Cell Proteomics* **14**, 1137-1147, doi:10.1074/mcp.O114.042259 (2015).

4 Punjani, A. & Fleet, D. J. 3D variability analysis: Resolving continuous flexibility and discrete heterogeneity from single particle cryo-EM. *J Struct Biol* **213**, 107702, doi:10.1016/j.jsb.2021.107702 (2021).

5 Scheres, S. H. & Chen, S. Prevention of overfitting in cryo-EM structure determination. *Nat Methods* **9**, 853-854, doi:10.1038/nmeth.2115 (2012).

6 Rosenthal, P. B. & Henderson, R. Optimal determination of particle orientation, absolute hand, and contrast loss in single-particle electron cryomicroscopy. *J Mol Biol* **333**, 721-745, doi:10.1016/j.jmb.2003.07.013 (2003).

7 Punjani, A., Rubinstein, J. L., Fleet, D. J. & Brubaker, M. A. cryoSPARC: algorithms for rapid unsupervised cryo-EM structure determination. *Nat Methods* **14**, 290-296, doi:10.1038/nmeth.4169 (2017).

8 Afonine, P. V. *et al.* Real-space refinement in PHENIX for cryo-EM and crystallography. *Acta Crystallogr D Struct Biol* **74**, 531-544, doi:10.1107/S2059798318006551 (2018).

9 Williams, C. J. *et al.* MolProbity: More and better reference data for improved all-atom structure validation. *Protein Sci* **27**, 293-315, doi:10.1002/pro.3330 (2018).

10 Kater, L. *et al.* Construction of the Central Protuberance and L1 Stalk during 60S Subunit Biogenesis. *Mol Cell* **79**, 615-628 e615, doi:10.1016/j.molcel.2020.06.032 (2020).

11 Hsieh, R. W. *et al.* Identification of ligands with bicyclic scaffolds provides insights into mechanisms of estrogen receptor subtype selectivity. *J Biol Chem* **281**, 17909-17919, doi:10.1074/jbc.M513684200 (2006).

12 Pettersen, E. F. *et al.* UCSF ChimeraX: Structure visualization for researchers, educators, and developers. *Protein Sci* **30**, 70-82, doi:10.1002/pro.3943 (2021).

13 Liu, X., Wang, Y. & Ortlund, E. A. First High-Resolution Crystal Structures of the Glucocorticoid Receptor Ligand-Binding Domain-Peroxisome Proliferator-Activated gamma Coactivator 1-alpha Complex with Endogenous and Synthetic Glucocorticoids. *Mol Pharmacol* **96**, 408-417, doi:10.1124/mol.119.116806 (2019).

14 Zhou, X. E. *et al.* Identification of SRC3/AIB1 as a preferred coactivator for hormone-activated androgen receptor. *J Biol Chem* **285**, 9161-9171, doi:10.1074/jbc.M109.085779 (2010).

15 Pei, J., Tang, M. & Grishin, N. V. PROMALS3D web server for accurate multiple protein sequence and structure alignments. *Nucleic Acids Res* **36**, W30-34, doi:10.1093/nar/gkn322 (2008).

16 Waterhouse, A. M., Procter, J. B., Martin, D. M., Clamp, M. & Barton, G. J. Jalview Version 2--a multiple sequence alignment editor and analysis workbench. *Bioinformatics* **25**, 1189-1191, doi:10.1093/bioinformatics/btp033 (2009).

17 Li, Y. *et al.* A mutant form of ERalpha associated with estrogen insensitivity affects the coupling between ligand binding and coactivator recruitment. *Sci Signal* **13**, doi:10.1126/scisignal.aaw4653 (2020).
