# Supplementary material for: Cryo-EM reveals the architecture of the PELP1-WDR18 molecular scaffold
Source: Nat Commun. 2022 Nov 9;13:6783. doi: 10.1038/s41467-022-34610-0 (PMC9646879; doi:10.1038/s41467-022-34610-0)
Supplement: Supplementary file 3 — Description of Additional Supplementary Files [file 41467_2022_34610_MOESM3_ESM.pdf]

Supplementary Data 1: This dataset includes the chemical crosslinks identified in the PELP1-WDR18 subcomplex and the Rix1 complex.

Supplementary Movie 1: Conformational heterogeneity observed within the N-terminal region of the PELP1 Rix1 domain.
