# Supplementary material for: Cryo-EM reveals the architecture of the PELP1-WDR18 molecular scaffold
Source: Nat Commun. 2022 Nov 9;13:6783. doi: 10.1038/s41467-022-34610-0 (PMC9646879; doi:10.1038/s41467-022-34610-0)
Supplement: Supplementary file 6 — Reporting Summary [file 41467_2022_34610_MOESM6_ESM.pdf]

nature research

Corresponding author(s): Robin Stanley

Reporting Summary

Nature Research wishes to improve the reproducibility of the work that we publish. This form provides structure for consistency and transparency in reporting. For further information on Nature Research policies, see Authors & Referees and the Editorial Policy Checklist.

Statistical parameters

When statistical analyses are reported, confirm that the following items are present in the relevant location (e.g. figure legend, table legend, main text, or Methods section).

n/a	Confirmed	
☐	☒	The exact sample size (*n*) for each experimental group/condition, given as a discrete number and unit of measurement
☐	☒	An indication of whether measurements were taken from distinct samples or whether the same sample was measured repeatedly
☐	☒	The statistical test(s) used AND whether they are one- or two-sided<br>*Only common tests should be described solely by name; describe more complex techniques in the Methods section.*
☒	☐	A description of all covariates tested
☒	☐	A description of any assumptions or corrections, such as tests of normality and adjustment for multiple comparisons
☐	☒	A full description of the statistics including central tendency (e.g. means) or other basic estimates (e.g. regression coefficient) AND variation (e.g. standard deviation) or associated estimates of uncertainty (e.g. confidence intervals)
☐	☒	For null hypothesis testing, the test statistic (e.g. *F*, *t*, *r*) with confidence intervals, effect sizes, degrees of freedom and *P* value noted<br>*Give P values as exact values whenever suitable.*
☒	☐	For Bayesian analysis, information on the choice of priors and Markov chain Monte Carlo settings
☒	☐	For hierarchical and complex designs, identification of the appropriate level for tests and full reporting of outcomes
☒	☐	Estimates of effect sizes (e.g. Cohen's *d*, Pearson's *r*), indicating how they were calculated
☐	☒	Clearly defined error bars<br>*State explicitly what error bars represent (e.g. SD, SE, CI)*

Our web collection on statistics for biologists may be useful.

Software and code

Policy information about availability of computer code

Data collection	Cryo-EM data was collected with SerialEM 4.0. Mass spectrometry data was collected with a Q Exactive Plus mass spectrometer (ThermoFisher Scientific) interfaced with an M-Class nanoAcquity UPLC system (Waters Corporation). ClairoStar automated plate reader was used to record luminescence data.
Data analysis	MotionCor2, CTFFIND4, CryoSparc_V3, UCSF Chimera_1.14, ChimeraX_1.4, Coot, Molprobity, Excel, Phenix, Protein Prospector (UCSF)

For manuscripts utilizing custom algorithms or software that are central to the research but not yet described in published literature, software must be made available to editors/reviewers upon request. We strongly encourage code deposition in a community repository (e.g. GitHub). See the Nature Research guidelines for submitting code & software for further information.

Data

Policy information about availability of data

All manuscripts must include a data availability statement. This statement should provide the following information, where applicable:

- Accession codes, unique identifiers, or web links for publicly available datasets
- A list of figures that have associated raw data
- A description of any restrictions on data availability

All data and plasmids used in this study will be made available upon request. EM maps and atomic coordinates for the cryo-EM model have been deposited in the EMDB (EMD-26831) and PDB (7UWF). Mass spectrometry data has been deposited in the Pride database.

Field-specific reporting

Please select the best fit for your research. If you are not sure, read the appropriate sections before making your selection.

☒ Life sciences ☐ Behavioural & social sciences ☐ Ecological, evolutionary & environmental sciences

For a reference copy of the document with all sections, see nature.com/authors/policies/ReportingSummary-flat.pdf

Life sciences study design

All studies must disclose on these points even when the disclosure is negative.

Sample size	The PELP1-WDR18 3D reconstruction was calculated from thousands of images. Sample size for all other experiments is provided in the figure legends.
Data exclusions	During cryo-EM data processing poor particle images were discarded following 2D and 3D classification.
Replication	Each IP/western blot was carried out at least three times. We performed both techincal and biological replicates of the luciferase assay. Detailed information of replicates is provided in the corresponding figure legends.
Randomization	Randomization was not relevant for this study.
Blinding	Blinding was not relevant for this study.

Reporting for specific materials, systems and methods

Materials & experimental systems

n/a	Involved in the study
☐	☒ Unique biological materials
☐	☒ Antibodies
☐	☒ Eukaryotic cell lines
☒	☐ Palaeontology
☒	☐ Animals and other organisms
☒	☐ Human research participants

Methods

n/a	Involved in the study
☒	☐ ChIP-seq
☒	☐ Flow cytometry
☒	☐ MRI-based neuroimaging

Unique biological materials

Policy information about availability of materials

Obtaining unique materials	All plasmids used in this study will be made available upon reasonable request.

Antibodies

Antibodies used	Primary antibodies used: anti-FLAG (Sigma #7425), anti-HA (Invitrogen #26183), anti-GFP (Roche #11814460001), anti-MYC (Sigma #05-724), anti-Tubulin (Invitrogen #MA1-80017), isotype antibody (Millipore #12-370), PELP1 antibody (Bethyl Labs #A300-180A-M), ER alpha antibody (Millipore #06-935) and WDR18 antibody (Sigma #HPA050200).

Validation	This study only made use of commercial antibodies that were validated by the manufacturer for western blot applications. We further validated Epitope tag antibodies using cells with and with out tagged expression constructs as controls.

Eukaryotic cell lines

Policy information about cell lines

Cell line source(s)	Hek293FT (Thermo), MCF7 (ATCC), HepG2 (ATCC)
Authentication	Cells lines were not authenticated.
Mycoplasma contamination	Cell lines were not tested for mycoplasma contamination.
Commonly misidentified lines (See ICLAC register)	No commonly misidentified lines were used in this study.
